# Fairness as an Ethical Imperative in Dental Assessment: German Students’ Perspectives on Oral Examinations and Structured Assessment Formats

**DOI:** 10.3390/dj14100644

**Published:** 2026-10-05

**Authors:** Lisa Morlock, Sarah Rampf, Stefan Rupf, Leonhard Kowalczyk, Konstantin Schrader, Jana Jünger, Anna-Lena Hillebrecht

**Affiliations:** 1 Department of Periodontology and Operative Dentistry, University Medical Center, Johannes Gutenberg University, Augustusplatz 2, 55131 Mainz, Germany; 2 Department of Conservative Dentistry, Faculty of Medicine, University Hospital Heidelberg, Heidelberg University, Im Neuenheimer Feld 400, 69120 Heidelberg, Germany; sarah.rampf@med.uni-heidelberg.de; 3 Synoptic Dentistry, Medical Faculty, Saarland University, Kirrbergerstr. 100, Bldg. 73, 66421 Homburg/Saar, Germany; stefan.rupf@uks.eu; 4 Federal Association of Dental Students in Germany (bdzm), Chausseestraße 14, 10115 Berlin, Germany; president@bdzm.de (L.K.);; 5 Institut für Kommunikations- und Prüfungsforschung gGmbH (Institute for Communication and Assessment Research), 69123 Heidelberg, Germany; j.juenger@cares.institute; 6 Department of Prosthetic Dentistry, Center for Dental Medicine, Medical Center—University of Freiburg, Faculty of Medicine, University of Freiburg, Hugstetterstr. 55, 79106 Freiburg, Germany

**Keywords:** dental education, oral examinations, structured oral examinations, assessment ethics, procedural fairness, transparency, examiner variability, student perspectives

## Abstract

**Background/Objectives:** Oral examinations can assess clinical reasoning and professional judgement, but examiner-dependent variation and unclear expectations may compromise reliability, procedural fairness, and accountability. This study investigated German dental students’ perceptions of oral examinations and identified learner-informed priorities for fair, transparent, and ethically responsible assessment. **Methods:** This national cross-sectional mixed-methods study used a participatory approach to questionnaire development. The main online survey was distributed to undergraduate dental students through national student networks. Three supplementary questions were administered separately to participants at the 2026 National Conference of German Dental Student Representatives. Quantitative data were analysed descriptively. Free-text responses were examined using qualitative content analysis conducted collaboratively by two researchers. **Results:** The main survey included 368 students from 26 dental schools, representing 84% of German dental schools; the separate conference-based dataset comprised 168 participants. Students strongly endorsed clearly defined competencies required to pass (mean 4.74 ± 0.81), transparent assessment criteria (4.64 ± 0.87), and structured questions to promote consistent and fair assessment (4.42 ± 0.99). Conference participants perceived a substantial influence of examiners on grades (4.25 ± 0.81). The qualitative findings contextualised the quantitative results across four areas: fairness and examiner influence; transparency of expectations and criteria; structure and consistency; and psychological safety, feedback, and preparation. **Conclusions:** Students viewed fairness, transparency, and accountability as integral to assessment quality. Structured oral examinations may support more equitable assessment while preserving opportunities to evaluate clinical reasoning and professional judgement. Because qualitative coding was conducted collaboratively rather than independently, no formal inter-rater reliability coefficient was calculated, limiting the reproducibility of the qualitative findings.

## 1. Introduction

Assessment is a central component of competency-based dental education. Beyond its traditional function of certifying achievement, it substantially influences students’ learning behaviour, motivation, professional identity formation, and the development of clinical competence [1]. Contemporary educational concepts therefore regard assessment not merely as a tool of learning but increasingly as a tool for learning, emphasizing transparency, meaningful feedback, and constructive alignment between learning objectives, teaching activities, and assessment strategies [2,3,4]. The transition towards competency-based dental education and the increasing adoption of Entrustable Professional Activities (EPAs) have further heightened the need for assessment methods that provide valid evidence of learners’ readiness for independent clinical practice [5,6]. High-quality assessments are essential not only for supporting learning but also for defensible high-stakes decisions and safeguarding patient safety [7].

Oral examinations have traditionally played an important role in dental and health professions education worldwide [8,9]. In contrast to written examinations, they allow examiners to assess clinical reasoning, professional judgement, communication skills, and the application of knowledge in complex and authentic contexts [10]. However, concerns regarding the reliability, validity, and fairness of traditional oral examinations have led to more standardized assessment formats [11]. Structured oral examinations (SOEs) are standardized oral assessments based on predefined questions and expected answers, which are developed and internally reviewed in advance to ensure consistency and comparability across candidates. Such an approach has been successfully implemented in medical education, with Caldwell et al. demonstrating that a standardized oral examination enabled the assessment of clinical knowledge among medical students during a surgical clerkship [12]. In contrast to traditional oral examinations, which may allow greater variability in questioning and assessment, SOEs have emerged as a promising alternative, as they have the potential to assess clinical reasoning, decision-making, and knowledge integration in authentic clinical scenarios while offering greater standardization, reliability, and validity than traditional oral examinations [13].

Limitations of traditional oral examinations have been attributed to variability questioning strategies, examiner behaviour, and assessment standards, which may result in inconsistent performance evaluation and perceptions of unfairness among learners [14]. These concerns have been documented across medical and dental education and have prompted calls for more standardised and evidence-informed assessment approaches [15].

To address these limitations, SOEs have been introduced in health professions education programmes. By incorporating blueprinting, predefined assessment criteria, standardised questioning, and explicit performance expectations, SOEs aim to enhance assessment quality while retaining the educational benefits of oral examination formats [16]. Previous research suggests that structured assessment approaches may improve reliability, fairness, and acceptance among both examiners and learners [17]. Consequently, the implementation of structured assessment has become an important topic within contemporary competency-based health professions education. Assessment quality also has an ethical dimension. Examiner-dependent variation may affect equal treatment, procedural fairness, and accountability. Transparent criteria, comparable conditions, and respectful interactions are therefore essential not only for reliability but also for ethically responsible assessment.

Alongside psychometric quality, learner perspectives are increasingly recognised as an essential dimension of assessment quality. Modern assessment frameworks emphasise that assessments should not only generate valid judgements of competence but also support learning, foster motivation, and create educationally meaningful experiences [18,19]. Students’ perceptions of fairness, transparency, psychological safety, and alignment between teaching and assessment may therefore influence both examination performance and the educational impact of assessment [20]. SOEs can additionally assess ethical competence by requiring students to recognise ethical conflicts, balance competing principles, and justify clinical decisions. They may serve both as an ethically responsible assessment format and as a method for evaluating ethical reasoning in dental education. In addition, the increasing use of generative artificial intelligence (AI) raises concerns about the authenticity of students’ performance in written assessments. SOEs may help address these concerns by requiring students to demonstrate their knowledge and clinical reasoning directly under controlled examination conditions, thereby reducing opportunities to rely on AI-generated content. SOEs may represent a complementary assessment approach in the era of AI [21,22].

Although oral examinations continue to play a central role in dental education internationally, evidence regarding students’ experiences and expectations remains limited. In particular, little is known about which characteristics students perceive as essential for fair and effective oral examinations and to what extent these expectations correspond to established principles of structured assessment.

Given the ongoing implementation of the new German Dental Licensure Act, evidence on students’ perceptions of oral examinations may contribute to the further development of competency-based assessment in undergraduate dental education [9]. The results may provide a foundation for future participatory approaches that actively involve students in the design and evaluation of oral examinations. This nationwide survey aimed to explore dental students’ experiences with oral examinations and to identify learner-informed priorities for their future development. Specifically, the study addressed the following research question: How do dental students perceive oral examinations with regard to procedural fairness, transparency, examiner influence, structure, psychological safety, and overall assessment quality, and which priorities for improvement do they identify?

## 2. Materials and Methods

### 2.1. Study Design

This national cross-sectional study employed a convergent mixed-methods design to investigate undergraduate dental students’ experiences with oral examinations in Germany. Between March and April 2026, quantitative questionnaire data and qualitative free-text responses were collected simultaneously through a main online survey. In addition, three supplementary questions were completed digitally by participants attending the 2026 National Conference of German Dental Student Representatives in April 2026, generating a separate conference-based dataset. The datasets were analysed separately before being integrated during interpretation. The study followed the principles of participatory educational research by actively involving dental students in the development of the survey instrument and the interpretation of its practical implications.

### 2.2. Participatory Research Approach

This study was designed according to the principles of participatory educational research, recognising undergraduate dental students not merely as study participants but as experts in their own learning and assessment experiences. Students were actively involved in the conception of the study, the identification of relevant research questions, and the development of the survey instrument. Their experiences with oral examinations informed the selection and wording of questionnaire items to ensure that the survey addressed issues considered meaningful and relevant from the learner perspective.

Rather than evaluating students as passive recipients of assessment, the study sought to capture their perspectives as key stakeholders in the continuous development of assessment practices. This participatory approach aimed to strengthen the relevance, content validity and practical applicability of the findings while promoting learner-centered educational research. The results of the survey subsequently informed the development of a participatory educational intervention in which dental students actively contribute to the design, implementation and evaluation of structured oral examinations.

### 2.3. Survey Development

The questionnaire was developed specifically for this study by an interprofessional working group comprising experts in dental education, health professions education, and dental students (A.-L.H., S.R. (Sarah Rampf), S.R. (Stefan Rupf)., L.M., K.S., L.K.). Students were actively involved in identifying relevant research questions, reviewing item wording for clarity and comprehensibility, and prioritising assessment-related topics. The questionnaire content was informed by established quality dimensions of assessment and principles of competency-based assessment. Prior to distribution, the questionnaire was reviewed within the working group to identify and resolve potential ambiguities in wording and content. The questionnaire was developed as an exploratory instrument to capture students’ perceptions and experiences of oral examinations rather than as a psychometric scale. Accordingly, no formal pilot study or psychometric validation was conducted.

The final survey included items addressing transparency of examination requirements and procedures, perceived fairness, degree of examination structure, awareness and acceptance of structured oral examinations, perceived relevance for future professional practice, quality of feedback, emotional burden, and opportunities for preparation and practice. In addition, participants were invited to provide free-text comments regarding positive experiences, perceived challenges, and suggestions for improvement.

Most closed-ended items were rated on a five-point Likert scale ranging from 1 (“strongly disagree”) to 5 (“strongly agree”), while selected questions used dichotomous (yes/no) response options (Supplementary File S1).

### 2.4. Data Collection and Participants

The survey was administered anonymously online using Google Forms (Google LLC, Mountain View, CA, USA; https://docs.google.com/forms/d/e/1FAIpQLScvjEWXOmUyz9CefCgsowMKNEvUhB4_XYUy3s02OrSeqUB1pQ/viewform; accessed on 22 February 2026). The survey link was distributed through student networks, including the Federal Association of Dental Students in Germany (Bundesverband der Zahnmedizinstudierenden in Deutschland e.V., bdzm), local student councils, and university mailing lists. As the survey link was disseminated through these networks rather than sent directly to a defined list of individual students, the total number of students who received the invitation could not be determined. Consequently, a response rate could not be calculated. Recruitment was voluntary, and no direct or individualised invitations were sent by the investigators. No formal sample size calculation was performed, as a voluntary, non-probability sampling approach was used; therefore, the resulting sample should be considered a convenience sample rather than statistically representative of undergraduate dental students. In addition to the online survey, three supplementary questions were administered digitally during the 2026 National Conference of German Dental Student Representatives in April 2026 using the same survey methodology. These questions focused on the perceived influence of examiners on grading, alignment between teaching and assessment content, and the prioritisation of potential measures to improve the quality of oral examinations. Responses to these supplementary questions were analysed and reported separately. All undergraduate dental students were eligible to participate, irrespective of their year of study. To preserve anonymity, the year of study was not collected; therefore, potential differences in responses according to students’ level of training could not be assessed.

### 2.5. Data Analysis

Quantitative data were analysed descriptively using frequencies, percentages, means, and standard deviations as appropriate. Questionnaire items were analysed individually rather than combined into composite scale scores. Qualitative data consisted of free-text responses to open-ended questions and were analysed using qualitative content analysis following the principles described by Mayring [23]. Responses were systematically coded and grouped into thematic categories reflecting recurring patterns in students’ experiences, perceptions, and recommendations regarding oral examinations. Coding and category development were conducted collaboratively by two researchers, with discrepancies discussed and resolved by consensus. Because coding was conducted collaboratively rather than independently, inter-rater reliability was not formally quantified.

### 2.6. Ethical Considerations

This study was conducted as an anonymous educational evaluation project involving undergraduate dental students. No patient data, clinical interventions, demographic information, or sensitive personal data were collected. Participation was entirely voluntary and anonymous, and no participant was in a direct supervisory, assessment, or dependency relationship with the investigators. According to institutional regulations for educational evaluation and quality assurance projects, formal ethical approval was not required. The Ethics Committee of the University of Freiburg confirmed that the study did not require formal ethical approval (reference no. 24-1406). Participation was voluntary and anonymous; completion of the questionnaire was considered to indicate informed consent for participation and data analysis.

## 3. Results

A total of 368 dental students from 26 dental schools across Germany participated in the survey, representing 84% of all dental education institutions nationwide. At the individual level, 2.3% of the undergraduate dental student population participated (Table 1, Table 2, Table 3 and Table 4). In addition, 168 participants responded to three supplementary questions administered during the 2026 National Conference of German Dental Student Representatives. The results of these questions are reported separately in Table 5 and Table 6.

### 3.1. Descriptive Statistics of Students’ Perceptions and Expectations Regarding Oral Examination

Students consistently emphasised transparency, explicit competency expectations and structured assessment procedures as the defining characteristics of high-quality oral examinations (Table 1). The highest agreement was observed for the statement that competencies required to pass the examination should be clearly defined (mean 4.74 ± 0.81), followed by clearly communicated assessment criteria (4.64 ± 0.87) and explicitly defined examination requirements (4.65 ± 0.92). Likewise, students strongly agreed that structured examination questions contribute to fairer assessment (4.42 ± 0.99).

In contrast, familiarity with structured oral examinations was comparatively limited (2.93 ± 1.28), despite widespread support for the principles that characterise structured assessment. Oral examinations were also associated with considerable uncertainty (3.43 ± 1.33), whereas students clearly valued individual feedback following examinations (4.27 ± 0.99).

**Table 1:** Results of items 1.1–1.13 presented as mean scores and standard deviations. Responses were rated on a five-point Likert scale ranging from 1 (“strongly disagree”) to 5 (“strongly agree”).

Item	Mean	SD
1.1 How clearly structured and comprehensible were the oral Z1 examinations for you?	3.10	1.18
1.2 How clearly structured and comprehensible were the oral Z2 examinations for you?	2.46	0.98
1.3 I consider it important that the procedure of an oral examination is clearly structured.	4.48	0.98
1.4 I consider it important that the requirements of an oral examination are clearly defined.	4.65	0.92
1.5 I would like to know in advance which criteria will be used for assessment.	4.64	0.87
1.6 It should be clear which competencies I must demonstrate to pass the examination.	4.74	0.81
1.7 Oral Z1 examinations should primarily assess theoretical competencies.	3.96	1.03
1.8 Oral Z2 examinations should primarily assess clinical competencies.	3.93	1.01
1.9 Clearly structured examination questions help ensure that all students are assessed consistently and therefore more fairly.	4.42	0.99
1.10 Oral examinations should contribute to preparing me for my future professional practice as a dentist.	4.20	1.10
1.11 Oral examinations are associated with a high degree of uncertainty for me.	3.43	1.33
1.12 I would like to receive specific feedback after the examination.	4.27	0.99
1.13 I am familiar with the format, procedures, and objectives of structured oral examinations (SOEs).	2.93	1.28

Abbreviations: SD = standard deviation; SOE = structured oral examination; Z1 = first section of the German dental licensing examination; Z2 = second section of the German dental licensing examination.

### 3.2. Students’ Perceptions of Assessment Practices at Their Dental Schools

Most students (70.4%) reported that SOEs were not used in regular teaching sessions or practical courses at their dental schools, while written examinations remained the dominant assessment format (68.8%) throughout the curriculum (Item 1.15) (Table 2).

**Table 2:** Distribution of responses to items 1.14–1.15, presented as frequencies and percentages.

Item	Response Option	Frequency (*n*, %)
1.14 Structured oral examinations (SOEs) are also used in teaching sessions and/or practical courses at my dental school.	Yes	109 (29.6%)
	No	259 (70.4%)
1.15 Student performance in courses and practical training is assessed predominantly through written examinations.	Yes	253 (68.8%)
	No	115 (31.3%)

### 3.3. Students’ Priorities for Improving Oral Examinations and Perceptions of Current Assessment Practices

In an open-ended question asking students which measures they considered most important for improving oral examinations, responses were subsequently coded into categories based on their content. Students clearly prioritised measures that promote fairness and consistency. Greater consistency between examiners was identified as the single most important improvement (29.1%), followed by better preparation materials and sample questions (23.4%) and transparent assessment criteria (17.4%) (Table 3). In contrast, feedback after examinations was selected less frequently as the primary improvement, suggesting that students currently perceive transparency and consistency as more pressing needs than post-examination reflection. Question 1.17 was another open-ended question, asking which assessment format predominated at participants’ dental schools. Of the 368 participants, 253 (68.8%) provided a response, while 115 (31.3%) did not respond. Among respondents, multiple-choice examinations were the most frequently reported coded category (186/253, 73.5%), followed by a combination of different assessment formats (42/253, 16.6%) and open-ended or short-answer examinations (24/253, 9.5%). One respondent (0.4%) indicated not knowing which assessment format predominated.

**Table 3:** Results of items 1.16–1.17 presented as absolute frequencies and percentages.

Item	Coded Category	Frequency (*n*, %)
1.16 What would be the most important improvement to oral examinations for you?	Greater consistency between examiners	107 (29.1%)
	Better preparation materials and sample questions	86 (23.4%)
	Clear assessment criteria provided in advance	64 (17.4%)
	Reduced examination-related stress and greater transparency of the examination process	59 (16.0%)
	Standardised examination structure and guidance framework	40 (10.9%)
	More feedback following the examination	12 (3.3%)
1.17 Which assessment format predominates at your dental school?	Multiple-choice examinations	186 (50.5%)
	A combination of different assessment formats	42 (11.4%)
	Open-ended or short-answer examinations	24 (6.5%)
	I do not know	1 (0.3%)
	No response	115 (31.3%)

### 3.4. Students’ Priorities Regarding Oral Examinations and Preferred Preparation Strategies

In the open-ended question (Item 1.18), students were asked to describe the key characteristics of high-quality oral examinations. Responses were subsequently coded into categories based on their content, with fairness, standardisation, transparency, and clear assessment criteria reflecting the key areas identified in the quantitative findings (Table 4). Regarding preparation, they expressed a strong preference for explicit learning objectives, transparent examination procedures, sample questions, opportunities for practice, and close alignment between teaching and assessment (Item 1.19). Notably, mock examinations and examination simulations ranked among the preferred preparation strategies, indicating a strong demand for assessment activities that familiarise students with authentic examination conditions.

**Table 4:** Results of items 1.18–1.19 presented as absolute frequencies and percentages.

Item	Coded Category	Frequency (*n*, %)
1.18 What is particularly important to you in oral examinations?	Fairness and equal treatment	58 (15.8%)
	Consistency and standardisation	52 (14.1%)
	Clear structure and transparency	49 (13.3%)
	A supportive examination atmosphere	41 (11.1%)
	Objectivity	24 (6.5%)
	Clear assessment criteria	22(6.0%)
	Alignment with the curriculum	21 (5.7%)
	Guidance and dialogic interaction during the examination	19 (5.2%)
	Feedback	13 (3.5%)
	Opportunities for preparation and practice	13 (3.5%)
	Clear and understandable questions	12 (3.3%)
	Flexibility in question wording	11 (3.0%)
	Assessment of clinical reasoning and understanding	10 (2.7%)
	Preparation time	7 (1.9%)
1.19 What would you like as preparation for oral examinations?	Clear learning objectives and explicit performance expectations	72 (19.6%)
	Sample questions	41 (11.1%)
	High-quality learning materials	37 (10.1%)
	Transparency regarding examination procedures and assessment criteria	31 (8.4%)
	Mock examinations or examination simulations	28 (7.6%)
	Clear structure and standardisation	22 (6.0%)
	Alignment between teaching and assessment	19 (5.2%)
	Revision courses	14 (3.8%)
	Early access to examination information	11 (3.0%)
	Opportunities for contact with examiners prior to the examination	10 (2.7%)
	Feedback	8 (2.2%)
	More opportunities to practise oral examinations during the curriculum	7 (1.9%)
	Psychological preparation and support	6 (1.6%)

### 3.5. Students’ Perceptions of Examiner Influence and Alignment Between Teaching and Assessment

Two of the three supplementary questions administered during the 2026 National Conference of German Dental Student Representatives addressed students’ perceptions of examiner influence and the alignment between teaching and assessment (Table 5). Students reported a high level of agreement that examination grades depend substantially on the examiner (4.25 ± 0.81) (Item 2.1). Nevertheless, students reported a generally positive alignment between the content taught in courses and that assessed in oral examinations (Item 2.2). These findings suggest that students primarily perceive variability in assessment practice rather than deficiencies in curricular alignment.

**Table 5:** Responses to items 2.1–2.2 regarding perceived examiner influence and alignment between teaching and assessment.

Item	Mean	SD
2.1 To what extent do you believe that the examiner influences your examination grade?	4.25	0.81
2.2 To what extent do you think the content taught in courses is aligned with the content assessed in oral examinations?	3.57	0.78

### 3.6. Quality Improvement Measures for Oral Examinations

The third supplementary question administered during the 2026 National Conference of German Dental Student Representatives asked students to prioritise measures for improving oral examinations (Table 6). Students most frequently selected clearly defined learning objectives (33.3%), followed by standardised examination questions (21.4%), mock examinations (17.3%), examiner training (15.5%) and structured assessment rubrics (12.5%). Together, these responses indicate a clear preference for structured, transparent and competency-oriented assessment approaches.

**Table 6:** Responses to item 2.3 regarding priorities for improving the quality of oral examinations, presented as frequencies and percentages.

Item	Response Option	Frequency (*n*, %)
2.3 Which of the following measures would most improve the quality of oral examinations?	Clearly defined learning objectives	56 (33.3%)
	Standardised examination questions	36 (21.4%)
	Mock examinations or examination simulations	29 (17.3%)
	Examiner training	26 (15.5%)
	Structured assessment rubrics	21 (12.5%)

### 3.7. Qualitative Findings

Qualitative content analysis complemented the quantitative findings and identified four overarching themes that consistently reflected students’ expectations regarding high-quality oral assessment.

**Fairness, equal treatment, and examiner-dependent variation**

First, participants frequently referred to fairness, objectivity, and examiner influence. They repeatedly emphasised the need for greater consistency among examiners, calling for “more comparability between examiners” and requesting that “all students should be treated equally and assessed according to the same criteria” (Item 1.16). One participant stressed that examinations should be conducted “according to the same standards and without personal sympathies influencing the assessment” (Item 1.18). Accordingly, fairness and equal treatment were the most frequently reported quality criteria among the coded responses.

**Transparency of expectations and assessment criteria**

Second, responses highlighted the importance of transparency of expectations and assessment criteria. Participants emphasised clear assessment criteria (Item 1.16), explicit performance expectations, and clearly defined learning objectives (Item 1.19). This was reflected in comments calling for “clearly structured questions, clearly defined topics, and transparent performance expectations”.

**Structure, standardization and consistency**

Third, participants stressed the value of structure, standardisation, and consistency. Standardised examination questions and structured assessment rubrics were identified as key measures to improve examination quality. One student stated, “Clearly defined requirements, so that students can be confident they will pass if they meet the expected standards”, highlighting the importance of predictable and transparent assessment procedures (Item 1.19).

**Psychological safety, feedback and preparation**

Finally, responses addressed psychological safety, feedback, and preparation. Participants highlighted the importance of psychological preparation and support, opportunities for preparation and practice, and constructive feedback before and after examinations (Items 1.18 and 1.19). For example, one participant explicitly requested “direct feedback from the examiner”, underlining the perceived value of timely and constructive feedback for learning.

## 4. Discussion

This study explored dental students’ experiences with oral examinations and identified learner-informed priorities for their future development. The findings suggest that students perceive oral examinations as an important component of dental education but express substantial concerns regarding fairness, transparency, and consistency. Beyond fairness, examiner-dependent variation may introduce construct-irrelevant variance, thereby undermining the validity of high-stakes decisions regarding clinical competence [11].

This observation is consistent with previous research showing that traditional oral examinations are susceptible to examiner-related variability, subjective judgement, and limited reliability [16,24]. The present findings confirm that these challenges remain highly relevant from the learner perspective. Although oral examinations were not identified as the predominant assessment format in routine dental education, they are of particular importance in Germany because all three state examinations required for dental licensure are conducted in an oral format. This discrepancy highlights the need for well-structured and standardised oral examinations, particularly in high-stakes assessment settings.

Rather than advocating the replacement of oral examinations, students expressed a clear preference for improving existing formats through greater transparency, clearer expectations, and enhanced standardisation. These preferences align with contemporary principles of high-quality assessment, which emphasise explicit assessment criteria, fairness, and assessment literacy [25]. The findings therefore support the continued relevance of oral examinations as an assessment format, while the literature highlights their potential to assess competencies such as clinical reasoning, professional judgement, and the application of knowledge in authentic clinical contexts [26,27]. These competencies were not directly assessed in the present study. Accordingly, oral examinations continue to occupy an important role within competency-based and programmatic assessment systems, provided that they are designed to promote fairness, transparency, meaningful feedback, and educational quality [28].

A central finding of this study is the close alignment between students’ expectations and the core principles of SOEs (Table 7). Students emphasised transparent assessment criteria, clearly defined learning objectives, standardised procedures, and comparable assessment conditions, closely aligning with established strategies to improve oral assessment quality, including blueprinting, standardised questioning, explicit performance criteria, and structured rating instruments [16,29]. The very high ratings for Items 1.3–1.6 and 1.9 (means 4.42–4.74) indicate broad endorsement of transparency, consistency, and structured assessment principles. However, the clustering of responses at the upper end of the scale suggests potential ceiling effects and may partly reflect socially desirable responding. Consequently, these findings support the general salience of these principles but do not permit a fine-grained ranking of their relative importance or precise conclusions regarding the strength of endorsement. These findings complement previous evidence that structured oral examination approaches improve reliability, validity, and acceptance while maintaining the educational strengths of oral assessment [16].

The ethical principles represent an interpretive mapping derived from the reported student priorities, survey evidence, corresponding SOE components, and intended outcomes; no additional empirical findings are introduced.

The priorities expressed by German dental students closely corresponded to internationally recognised quality criteria for structured oral examinations. Despite limited familiarity with SOEs (2.93 ± 1.28), students independently identified key elements that constitute evidence-based oral assessment, including transparency, standardisation, explicit performance expectations, examiner calibration, feedback, and structured preparation. This convergence suggests that learner expectations and contemporary assessment frameworks, which emphasise that high-quality assessment should support both valid and defensible judgements of competence and students’ learning, motivation, and professional development.

Interestingly, participants demonstrated only limited familiarity with structured oral examinations, yet consistently prioritised the very elements that define evidence-based SOEs. This finding suggests that students intuitively recognise the conditions required for fair and meaningful assessment. The close convergence between learner expectations and contemporary assessment theory strengthens the argument that structured oral examinations represent not merely a psychometric improvement, but also a learner-centred response to the challenges associated with traditional oral assessment formats.

The identified themes extend beyond oral examinations alone and reflect broader principles of assessment literacy and learner-centred, programmatic assessment [30]. Participants consistently valued transparent learning objectives, explicit assessment criteria, standardised assessment procedures, meaningful feedback, and opportunities for preparation. These preferences align with contemporary assessment frameworks, which emphasise that high-quality assessment should support both valid and defensible judgements of competence and learning, motivation, and professional development [25,31]. From this perspective, assessment quality is determined not only by the validity of individual assessment formats but also by the extent to which assessment practices promote transparency, fairness, learner engagement, and informed decision-making.

The findings also underscore the importance of assessment literacy. Contemporary assessment theory suggests that students benefit when they understand assessment standards, performance criteria, and the rationale underlying assessment decisions, thereby supporting self-regulated learning, effective use of feedback, and the development of evaluative judgement [32]. Notably, participants’ priorities including transparent learning objectives, explicit assessment criteria, sample questions, and structured feedback closely mirror the educational conditions considered essential for developing assessment literacy [33]. Rather than requesting less demanding examinations, students appeared to seek greater clarity regarding expectations and assessment processes. Furthermore, the importance attributed to psychological safety suggests that the quality of assessment should be considered not only in terms of measurement properties but also in relation to learners’ experiences within the assessment environment [34,35].

Another important finding concerns preparation for oral examinations. Students expressed a clear preference for explicit learning objectives, sample questions, opportunities for practice, and examination simulations. Previous studies have demonstrated that structured preparatory activities, including mock examinations, can improve examination performance and reduce assessment-related anxiety [36,37]. These findings support the integration of structured preparation opportunities and structured oral assessment principles earlier within dental curricula. Such approaches may enhance assessment literacy, reduce uncertainty associated with high-stakes examinations, and help students build confidence in communicating and applying their knowledge under assessment conditions. Although the overarching qualitative themes corresponded to the domains addressed by the Likert-scale items, the free-text responses provided contextual insights into students’ experiences and concerns, including specific examples, perceived challenges, and suggestions for improvement that could not be captured through the predefined response options. The qualitative findings therefore complemented and contextualised the quantitative results rather than constituting an independent source of evidence.

**Ethical implications of oral assessment**

From an ethical perspective, assessment quality cannot be reduced to reliability and validity alone. Examiner-dependent variation also raises questions of equal treatment, procedural fairness, and accountability. Transparent expectations, comparable conditions, structured procedures, and respectful interactions can make high-stakes decisions more transparent and defensible without eliminating professional judgement. Involving students in assessment development further reflects a commitment to participation and shared responsibility. When based on appropriately designed clinical cases, SOEs may also assess students’ ability to recognise ethical conflicts, balance competing principles, and justify ethically defensible decisions.

**Strengths and limitations**

The participatory design of this study further reflects the growing international movement towards students as partners in health professions education. Rather than viewing students solely as recipients of assessment, this approach recognises them as important stakeholders in the continuous improvement of educational practice. By actively involving students in the development of the questionnaire, the study not only explored learner perceptions but also incorporated student expertise into the research process itself. Such participatory approaches have been shown to improve the relevance, authenticity and educational impact of curriculum and assessment development. A strength of this study was the broad institutional coverage, with participants recruited from 26 German universities, representing 84% of dental schools in Germany. This broad representation across institutions enabled the identification of common themes across diverse educational settings. However, the proportion of participating students was approximately 2.3% of the total dental student population, and the number of students who received the survey invitation was unknown; therefore, a response rate could not be calculated. As oral examinations constitute a common assessment format across the health professions, the findings of this study may be applicable to other educational contexts and contribute to broader discussions on the design and quality assurance of oral assessments.

Several limitations should be considered. The study relied on self-reported perceptions and experiences, which may be influenced by individual examination experiences and response biases. In addition, recruitment through student representative networks and the voluntary nature of participation may have introduced selection bias, as students with a particular interest in assessment and examination-related issues may have been more likely to participate. Due to the anonymous nature of the data collection, it was not possible to determine whether participants in the main survey also participated in the supplementary questions administered at the conference; therefore, overlap between the two datasets cannot be excluded. Furthermore, anonymisation prevented stratification by year of study, institution, or prior SOE exposure, limiting the assessment of potential differences in students’ perceptions across educational stages and contexts. The questionnaire was developed as an exploratory instrument rather than as a psychometric scale and was therefore not formally psychometrically validated. Its content and comprehensibility were reviewed by an interprofessional working group prior to distribution. Response rates could not be determined. In addition, some question items may have been perceived as leading, potentially introducing response bias. Although qualitative coding was conducted collaboratively by two researchers, no formal inter-rater reliability coefficient was calculated, which may limit the reproducibility of the qualitative findings. Furthermore, the cross-sectional design precludes causal inferences, and no institution-specific analyses were conducted. The high ratings observed for several items (mean scores 4.42–4.74 on a 5-point scale) may indicate ceiling effects and the potential for socially desirable responding, which may have limited the ability to discriminate between participants’ perceptions. The high proportion of non-responses to Item 1.17 (31.3%) limits the strength of conclusions regarding the predominant assessment format and may have introduced non-response bias. Finally, the study focused exclusively on the student perspective. Future research should incorporate faculty perspectives and objective assessment outcomes to provide a more comprehensive understanding of the implementation and effectiveness of structured oral examinations.

**Implications for dental education**

The findings have implications that extend beyond the national context in which the study was conducted. Students’ priorities closely mirror internationally recognised principles of high-quality assessment, suggesting that similar challenges and opportunities may exist across dental education programmes. The implementation of structured oral examinations, transparent assessment criteria, examiner training, and learner-centred preparation strategies may contribute to more equitable, reliable, and educationally meaningful assessment practices internationally.

## 5. Conclusions

Dental students regard oral examinations as an important assessment format but identify substantial opportunities for improvement regarding transparency, fairness, consistency, and preparation. Notably, students advocate for many of the core design principles of structured oral examinations even when they are unfamiliar with the format itself. Greater standardisation, explicit assessment criteria, structured preparation, and examiner training may improve both the educational quality and perceived fairness of oral examinations. At the same time, such measures may help retain the potential advantages of oral assessment described in the literature, including opportunities to assess clinical reasoning and professional judgement. By promoting transparent procedures, comparable conditions, and accountable decision-making, structured oral examinations may also contribute to more ethically responsible assessment in dental education and, ultimately, support patient safety by helping to ensure that clinical competence is assessed more consistently.

## Figures and Tables

**Table 7:** Alignment between student-reported priorities, ethical principles, and components of structured oral examinations.

Student-Reported Challenge or Priority	Evidence from the Survey	Ethical Principle	Corresponding SOE Component	Intended Educational Outcome
Perceived examiner influence	Examiner influence on grading: 4.25 ± 0.81	Procedural fairness; accountability	Examiner training and calibration	Improved fairness and reliability
Need for transparency	Assessment criteria known in advance: 4.64 ± 0.87	Transparency	Explicit assessment rubrics	Greater transparency and trust
Need for clearly defined expectations	Competencies required to pass should be clear: 4.74 ± 0.81	Transparency; procedural fairness	Blueprinting and learning outcomes	Improved alignment and validity
Need for consistency	Consistency between examiners identified as top improvement (29.1%)	Equal treatment	Standardised questioning	Increased inter-examiner consistency
Need for structure	Importance of structured examinations: 4.48 ± 0.98	Procedural fairness; predictability	Structured examination framework	Reduced variability
Need for objectivity	Fairness and objectivity among most frequently mentioned priorities	Impartiality; accountability	Structured scoring instruments	More defensible decisions
Need for preparation	Learning objectives (19.6%), sample questions (11.1%), simulations (7.6%)	Equality of opportunity; procedural fairness	Mock SOEs and preparatory activities	Improved assessment literacy
Need for feedback	Desire for feedback: 4.27 ± 0.99	Respect for learners; educational responsibility	Assessment-for-learning strategies	Enhanced learning impact
Examination-related uncertainty	Uncertainty associated with oral examinations: 3.43 ± 1.33	Psychological safety; respect	Transparent procedures and practice opportunities	Reduced anxiety and increased psychological safety
Professional relevance	Relevance for future practice: 4.20 ± 1.10	Professional responsibility	Authentic competency-based assessment	Improved educational relevance

## Data Availability

The raw data supporting the conclusions of this article will be made available by the authors upon reasonable request, subject to applicable data protection and institutional requirements.

## Supplementary Materials

The following supporting information can be downloaded at https://www.mdpi.com/article/10.3390/dj14100644/s1. Supplementary File S1: Questionnaire.

## Abbreviations

The following abbreviations are used in this manuscript:

EPA	Entrustable Professional Activities
SD	Standard deviation
SOE	Structured oral examinations
Z1	first section of the German dental licensing examination
