# Supplementary material for: Fairness as an Ethical Imperative in Dental Assessment: German Students’ Perspectives on Oral Examinations and Structured Assessment Formats
Source: Dent J (Basel). 2026 Oct 5;14(10):644. doi: 10.3390/dj14100644 (PMC13703168; doi:10.3390/dj14100644)
Supplement: Supplementary file 1 [file dentistry-14-00644-s001.zip › dentistry-4475221-supplementary.pdf]

Supplementary File S1

Questionnaire:

S1. Item 1.1-1.13

Item
1.1 How clearly structured and comprehensible were the oral Z1 examinations for you?
1.2 How clearly structured and comprehensible were the oral Z2 examinations for you?
1.3 I consider it important that the procedure of an oral examination is clearly structured.
1.4 I consider it important that the requirements of an oral examination are clearly defined.
1.5 I would like to know in advance which criteria will be used for assessment.
1.6 It should be clear which competencies I must demonstrate to pass the examination.
1.7 Oral Z1 examinations should primarily assess theoretical competencies.
1.8 Oral Z2 examinations should primarily assess clinical competencies.
1.9 Clearly structured examination questions help ensure that all students are assessed consistently and therefore more fairly.
1.10 Oral examinations should contribute to preparing me for my future professional practice as a dentist.
1.11 Oral examinations are associated with a high degree of uncertainty for me
1.12 I would like to receive specific feedback after the examination.
1.13 I am familiar with the format, procedures, and objectives of structured oral examinations (SOEs).

The items were rated on a five-point Likert scale ranging from 1 (“strongly disagree”) to 5 (“strongly agree”). Abbreviations: Z1 = first section of the German dental licensing examination; Z2 = second section of the German dental licensing examination.

S2. Item 1.14-1.15

Item
1.14 Structured oral examinations (SOEs) are also used in teaching sessions and/or practical courses at my dental school.
1.15 Student performance in courses and practical training is assessed predominantly through written examinations.

The items were answered dichotomously (yes/no).

S3. Item 1.16-1.19

Item
1.16 What would be the most important improvement to oral examinations for you?
1.17 Which assessment format predominates at your dental school?
1.18 What is particularly important to you in oral examinations?
1.19 What would you like as preparation for oral examinations?

These questions were answered using free-text responses

S4. Item 2.1-2.3

Item
2.1 To what extent do you believe that the examiner influences your examination grade?
2.2 To what extent do you think the content taught in courses is aligned with the content assessed in oral examinations?
2.3 Which of the following measures would most improve the quality of oral examinations?

Item 2.1 was rated on a five-point Likert scale ranging from 1 ("not at all influential") to 5 ("strongly influential"). Item 2.2 was assessed using a five-point Likert scale ranging from 1 ("do not agree at all") to 5 ("completely agree"). Item 2.3 asked participants to prioritise the following measures to improve oral examinations: (A) clearly defined learning objectives, (B) structured marking rubrics, (C) standardised questions, (D) examiner training, and (E) mock examinations.
